# Reflections on the United States Military 1941-1987

**DOI:** 10.1186/1754-6834-2-20

**Published:** 2009-09-01

**Authors:** Mary Mandels, Douglas E Eveleigh

**Affiliations:** 1 Rutgers University, New Brunswick, NJ, USA

## Abstract

This article, 'Reflections on the United States Military 1941-1987' written by my grandmother, Mary Mandels, illustrates her passion for life. Her outreach article was considered most appropriate for publication in this forum. Her career activities are outlined in the prior article 'Mary Elizabeth Hickox Mandels, 90, Bioenergy Leader' while her accomplishments were fully recognized, for instance, nationally through the American Chemical Society and through her induction into the Hall of Fame at the US Army Soldier Systems Center in Natick, Massachusetts. As illustrated, along with Dr Elwyn Reese at Natick's Pioneering Research Laboratory, she headed a bioengineering group that is particularly remembered for developing a process for the enzymatic conversion of waste cellulosic biomass into soluble sugars that could be fermented to ethanol for an alternate liquid fuel (gasohol). This technology remains a subject of interest with growing environmental concerns and an oil shortage crisis.

Mary broke the promotional glass ceiling in her own field, all the more remarkable from the perspective that she was born 3 years before women gained the right to vote. Her talents as the family storyteller, enthralling her four siblings while growing up, later reflected her abilities as an outstanding mentor to young scientists. Mary's passions went beyond her career with a love of nature and the outdoors, taking frequent canoe, hiking, skiing, and camping trips. She had a broad fascination for science, foci including her encyclopedic knowledge of plants and wildlife. When not outdoors Mary enjoyed listening to music, from opera to 'Bobby' Dylan, as she called him. Her voracious appetite for books was apparent by the tomes that covered her coffee table. She was never shy to share her political opinions and would send long handwritten letters to politicians who did something to her disapproval. She was strong willed and passionate in everything that she did. In particular was her love of the nation and of the US Army, and this particular article reflects her passion. Mary was an inspiration to all of those who knew her. For me, she was not only my grandmother but also my friend and role model. I will forever miss her wisdom, spirit and passion for life.

Susan Roche

## Commentary

What ails the United States military establishment? Few even in government or military circles would deny that problems exist. Since World War II our only unequivocal military victory was the invasion of Grenada where we defeated a few hundred Cuban construction workers. Since I have enjoyed a fulfilling position in an army research laboratory for more than 30 years, the question troubles me greatly. Here I comment from the perspective of nearly 50 years personal observation, first as the wife of an army officer who later became a civilian employee of the army, and second my own long-term service. Much of what I have to say is personal opinion, anecdotal, and based on observations of one small laboratory (annual budget about $70,000,000) not even involved with weapons. But as the whole is the sum of its parts, the deficiencies of an institution too large to be comprehensible to a single human being can perhaps best be understood by observation of the components. The obvious problem is well meaning but incompetent management resulting in inefficiency, waste, and cover up. But I believe the fundamental problems are more serious and reflect arrogance, malaise, and a decline in morality of American society and government.

It was not always so! I was born in 1917 (3 years before women could vote) and so attended school in the 1920s. In those halcyon days the army was remote from civilians except for romantic and tragic memories of World War I and other previous wars. We had a War Department that was really for defense because, as our teachers assured us, the United States would never again become involved in an overseas war. World War I had killed their sweethearts or potential husbands and condemned the teachers to lives as spinsters. It had created most of the problems we now saw in Europe and other distant parts of the world. The villains were war profiteers and international cartels of munitions manufacturers. The United States was always right, always noble, we had never lost a war and we never would. The army was small, professional, and idle. There was no draft, no registration of 18 year olds, and the only soldiers we ever saw were in parades on *Armistice Day *and *Decoration Day*. Then we bought poppies and forget-me-nots to help the wounded veterans and a soldier would come to school to tell us about our brave heroes and our great country. When I was very young the soldier was an ancient Civil War veteran, later we were visited by middle aged Spanish American War or younger khaki clad World War I veterans.

In the 1930s came the Depression, the New Deal, and Hitler. World War II came to Europe in 1939 and to the United States in 1941. We were still largely a rural and unsophisticated nation. We had no enthusiasm for this war, but we were sure we were right and we knew we would win, and of course we did, but in so doing, we irrevocably changed our lives and the character of our country. The war dominated our lives. My father ate supper with the radio at his ear and no one could interrupt it. The daily papers had pictures of pathetic refugees fleeing the advancing armies and alarming maps of the territories falling to the Germans and the Japanese. The country plunged into war production, food and gasoline rationing, and shortages. No new cars were built for the duration. All the factories were producing planes, tanks, and munitions. Everyone was in military service, war production, or agriculture. My father was a superintendent in a munitions plant. My brother was with Bradley's army in Europe. My husband was with Curtis Le May's B-29s in Guam and Saipan. My brother-in-law was with Patton's army in Europe. My sister was a Wave with the Navy in Washington. Everyone travelled [*sic*]. Boys who had never expected to leave home went off to training camps, usually in the south, and then overseas. Young wives followed their husbands as long as possible. I took the long train ride from the Northeast to Texas with a baby daughter. It was a shock to a liberal notherner [*sic*] to see the 'Whites Only' and 'Colored' signs on waiting room, rest rooms, and drinking fountains, and to ride on buses where the blacks had to sit in the rear. In northern towns in those days there were almost no blacks and so, little discrimination. There was one middle class black family in my grammar school district. Their children, two well scrubbed little girls in pigtails, attended school with us, played with us, and we looked on them with greater favor than we did the unwashed Italian and Polish immigrants that flooded our factory town. But that was all to change as blacks streamed north to work in defense industries and joined the army to fight alongside whites. Women were also going to work in defense industries and the military services, and replacing men in professional jobs such as college instructors. It would never again be the same white man's world.

In April 1 1945, Germany's surrender ended the war in Europe. The Allies had won, thanks to the material and manpower from the United States and the uneasy alliance with Communist Russia. Europe was a devastated ruin and the horrors of the concentration camps were revealed to a shocked world. In August, atomic bombs were dropped on Hiroshima and Nagasaki and the Japanese surrendered. For a brief sweet moment the United States was the unchallenged number one industrial, military, and financial world power. We did not doubt that we deserved this pre-eminence and had earned it by our hard labor and moral and intellectual superiority. The boys came home and were welcomed as heroes, but the military did not fade back into its pre-war obscurity. We had decided that our abrupt withdrawal from Europe, and isolation after World War I had been a mistake. This time we would stay long enough to shore up democracy and put the world in proper order. Besides, we did not trust our Russian allies who would rapidly move in wherever we withdrew.

So we joined in setting up the United Nations, but we also encouraged discharged soldiers to stay in the reserves, and the draft continued because occupation forces and military bases all over the world required a large army, navy, air force, and marine corps. Some of the citizen-soldiers liked army life and stayed in the services. The officers among these were more interested in management operations and career prerequisites and less interested in military tactics than were the professional soldiers who had led the military during and before the war. They also differed from those citizen-soldiers who disliked the rigid chains of command and unquestioning obedience expected in the services and who returned as fast as possible to civilian life. There was a definite selection of organization types over more independent mavericks. In the 1950s and 1960s many of these new officers became colonels and generals and so played major roles in shaping military management and strategy.

Both my husband and myself were microbial physiologists in graduate school when the war came. We had hoped for academic careers in research and teaching. Before the war, our prospects were not bright. Such jobs were scarce, pay was low, and we had two strikes against us. He was a Jew, and I was a woman. Both of us had been advised not to plan on academic careers. But after World War II, a golden age for science arrived in the United States. Science was the 'Endless Frontier' generously supported by a public grateful for the technological miracles such as radar, penicillin, and the atomic bomb that had played such a role in winning the war. The military establishment became a major patron of science through channels such as the Office of Naval Research, and provided broad and generous support of fundamental research in universities, research institutes, industry, and new military laboratories. My husband went to work for the Quartermaster Corps, which set up a Pioneering Research Laboratory to investigate fundamental problems in Biology, Chemistry, and Physics relevant to military interests. Later I went to work for the same laboratory. It had a wonderful research atmosphere. Salaries were good and there was plenty of money for equipment, supplies, and technical assistance.

We had excellent administrators, even including the commanding general, who believed in basic research and so gave us leeway in selecting problems, and support in investigating them. Our colleagues were competent young scientists, many of them army veterans about our own age. This was a new and expanding effort, so young scientists were promoted rapidly and new younger scientists continued to be added to the roster. We were encouraged to publish, and to attend meetings. We scarcely realized that we were working for the army. It was too good to last!

At its peak, the Pioneering Research Laboratory had a staff of about 100 civilians. The larger Quartermaster installation of which we were a section had about 1500 civilians, mostly in product laboratories, and 100 military. The military officers were mostly administrative: commanding officer, executive officer, intelligence officer, comptroller, military liaison, and so on. We also had a headquarters company of enlisted men who served as aides to the officers, drivers, cooks, and so on. and some who had scientific training served as laboratory aides. Some of these were excellent, a few even had PhDs. They were, of course, draftees, counting the days until their release. The regular army volunteers were a different lot. Some planned a military career. Others were lost souls hoping through the army to escape their personal or environmental demons. The installation mission was research and development in support of the individual soldier: food, clothing, shelter, aerial delivery, effects of hostile environment (desert, arctic, high altitude, jungle). We did no weapons research or development.

Meantime, the country was enjoying peace and prosperity to the hilt. Most of us had never had it so good. Suddenly everyone had a car and a house of their own. The men had good jobs and, for the moment, women and blacks were content or at least silent. The Marshall Plan was rebuilding Europe, including Germany. MacArthur was converting Japan to a successful industrial democracy. Americans were proud of themselves. We had saved our friends and made friends with our enemies.

The only flaw was our deteriorating relationship with Russia, our wartime ally. Germany was divided and Eastern Europe was now behind the 'Iron Curtain'. When in 1949 the Communists took over China and the Russians exploded an atomic bomb, our euphoria evaporated. We were no longer the unchallenged military superpower. This was a terrible psychological shock to the American people. We were so ready to lead the world that it had not occurred to us that the world might not be ready to follow. So began the Cold War. We developed the hydrogen bomb but, all too soon, the Russians had a better one. Surely someone had betrayed us and stolen our technological secrets! A witch hunt for scapegoats culminated in Joseph McCarthy.

Korea was a new kind of war, neither declared nor won. We avowed we would protect democracy there but the Communists (with some Chinese assistance) drove us out of North Korea, and we ended up supporting a right-wing dictatorship in South Korea, which required maintaining American troops there for 35 years now, with no end in sight. It has always been ambiguous whether those troops are to protect the South Korean dictatorship from the Communists or from the will of their people.

When the Russians launched Sputnik in 1957, we could not blame this on stolen secrets. Many of the former German rocket scientists were working in our laboratories, but we could not even put a man in space, let alone in orbit. Now we were not even number one in scientific technology! President Kennedy launched the 'Space Race' and by 1969 we had put a man on the moon and declared that we had won. But had we, or was the moon landing largely a public relations spectacular? We don't go there anymore. Russian progress in such aspects of space science as planetary exploration and long duration space flight is still more impressive than our own. Our shuttle program is in collapse and has always lacked clear scientific or commercial objectives. Today, our major objectives in space are military.

The cold war and the space race did lead to increases in support for science education and scientific research, but this was no longer the unqualified trusting support of the early post war years. Despite all that support, we had let the Russians get ahead of us. The cost of research was rising rapidly. Salaries were up, equipment was more expensive, and there were an ever-increasing number of projects. Competition for money increased, and with the Vietnam War, real growth in funds for scientific research ended. Now much of the money was earmarked for specific military or space hardware. Money for fundamental research was more grudgingly distributed. Clear relevance to some military or practical problem had to be demonstrated.

The inevitable result was the rise of management. In 1960 President Kennedy appointed Robert McNarnara Secretary of Defense. He was a systems analyst who had risen to be the much-admired president of the Ford Motor Company. Now he was determined to establish civilian control of the Pentagon. His 'whiz kids' installed computers and computerized systems to manage and keep track of everything and to bring order and sound fiscal management to the chaotic and bloated military establishment. A quarter of a century later, the Pentagon is more chaotic and bloated than ever, and the American automobile industry is no longer regarded as a paragon of good management. What went wrong? McNamara was an unusually capable, intelligent, and well meaning individual who was highly successful at getting people to believe him and to carry out his wishes, even when he was absolutely wrong. Systems analysis is a methodology for quantifying problems and dividing them into manageable sections, and is indeed a great help in achieving objectives. It does not concern itself with the morality or common sense of the objectives. The automobile industry was very successful at turning out big expensive cars that were profitable as long as there was no alternative. Profits faded when the Japanese demonstrated that many consumers preferred to buy small, economical and reliable vehicles. We poured billions of dollars, megatons of equipment, and hundreds of thousands of American troops into Vietnam in a futile attempt to force an unwanted government on an unwilling populace. The American interest there was never clearly or consistently explained. In the end, our involvement wounded us, devastated Vietnam, and established Communism in the reunited country. It caused no discernible damage to our 'real enemies' China and Russia, and our defeat did not lead to the predicted triumph of Communism in the 'domino' nations. Communism is not a way of life that appeals to the observer in a contented society. It takes root successfully only in the poverty, misery, and hopelessness of a nation devastated by war (China, Vietnam) or right-wing tyranny (Russia, Nicaragua).

When McNamara's enthusiasm for the Vietnam War faded, President Johnson appointed him President of the World Bank, where he presided over a large expansion of its resources and the funding of many ambitious development projects in the Third World. Today the bank is accused of supporting big dams that flooded rich agricultural lands and displaced large populations, and of clearing irreplaceable tropical forests for dubious agricultural enterprises such as cattle ranches that produce beef and vegetables not for native populations, but for export, often to countries that already have surplus. Environmentalists and Third World citizens complain that much of this well intentioned development benefited speculators, developers and the already wealthy, but increased misery, hunger and poverty in the indigenous people. For all three institutions (the automobile industry, the Pentagon, the World Bank) management was outstanding in achieving its immediate objectives, but unfortunately the longer range implications were insufficiently considered.

The wound that Vietnam inflicted on the United States military establishment including its civilian employees was to our motivation and self-image. In World War II, the objective was to win the war, and there were very few moral reservations about achieving this objective by any possible means. In the immediate post war years we continued to feel very good about ourselves. The military wanted to build a modern army and to defend freedom all over the world. The civilians wanted to give them better weapons, food, clothing, and shelter. The research scientists looked for critical problems and found interesting research areas to pursue with zeal and satisfaction.

By the 1960s serious doubts about some American policies were being expressed by the antiwar protestors and even a few respectable statesmen. The politicians who implemented the policies defended them strongly and the military joined forces with conservatives in a strident attack on the patriotism, intelligence, and the morality of the doubters. They protested too much. The attack was so strident because the attackers had to suppress their own growing uneasiness. Most of all they needed to convince themselves. Were we really fighting for peace and freedom or were we thinking of our own jobs, security, and power? President Eisenhower was right when he warned us about the Military-Industrial Complex. Today there is an alliance between politicians, the defense industry including the workers, the military including its civilian employees, and the vast research enterprise supported by the military. Peace and disarmament would cause painful readjustments. People want to be needed and they need to feel that what they are doing is worthwhile and important. Most people like their jobs, and even the ones who don't, do not wish to lose them. Military installations, think tanks, and the Defense industry provide jobs and money to localities all over the country. Even the peaceniks protest when the establishments in their localities are threatened.

So, although we do not admit it, least of all to ourselves, since Vietnam too much of our motivation is selfish. The major wishes to be a colonel, the scientist wants security and increased funding for his research project, the administrator wants his little empire to grow, the aerospace engineer wants to develop his intricate and marvelous machine, and the defense worker wants to continue his high pay and liberal overtime. The contractor wants to build a new and profitable installation, and the politician wants that installation to be in his district so that his happy constituents will re-elect him. It is most agreeable to work directly or indirectly for the government where a profit does not have to be made, where money flows freely, accountability is lax, and mediocrity is protected. But deep down, President Carter's malaise troubles us, much as we wish to agree with President Reagan that America feels good about itself.

Is Communism really such an ogre? Russia is a threat to us because it is a big expansionist nation, but we manage to coexist without war and are even happy to sell them subsidized grain. After 30 years of violent rhetoric, China is almost a friend. Small countries are another matter. Do we really have a right to go in and devastate small, weak countries to 'save them from Communism' even when that means inflicting them with a corrupt dictatorship? Why did we invade Grenada, but stand aside when Czechoslovakia and Hungary fell? Why did we bomb Libya, but not Iran or Syria? Have we become bullies who talk big but only pick fights with little guys? Maybe we began to lose credibility when the War Department became the Defense Department and perhaps it was all gone when President Reagan renamed the MX missile 'The Peacekeeper'. Does anyone really believe that 'Star Wars' is only for defense, or that we plan to share the technology with the Russians? For most of us such questions are strongly repressed.

In times of national emergency everyone pitches in a fairly selfless manner to solve problems that may be difficult, but are usually urgent and well defined. One cannot expect patriotic fervor to sustain people for 40 years in the absence of clearly visible threats. Naturally both military and civilian employees began to consider pay, working conditions, and career advancement.

During World War II, the Quartermaster product laboratories benefited from the services of technical experts on loan from the food and clothing industry. With the aid of a hastily assembled young staff they performed miracles in designing and producing new rations, fabrics, and needed items of clothing and shelter. After the war the experts returned to industry and their assistants took over the product laboratories. Frequently they retained the services of their mentors by hiring them as consultants or contracting out work to them. So now the former assistants were the managers. Everyone was making more money and, since the problems were no longer pressing, working at a more relaxed pace. In fact there was a bit of a scramble and competition to come up with problems of sufficient interest to retain funding and keep the laboratories in business.

So in the 1970s and 1980s the character of our laboratory changed, reflecting similar changes throughout the military establishment and its satellite enterprises. Even the academic world shows many of these changes. Previously the laboratory administration had provided support to the scientists. They had their own administrative budget. Now they were managing us and we provided their support through overhead charged to each research project. The new system was a blank check for management since they could set the overhead percentage. This grew rapidly as administrative staff and functions increased. The cost of supporting a scientific project was up, but the scientific staff was shrinking because there was not enough money left to hire young professionals and technicians to replace personnel who retired or resigned. The remaining staff was ageing and had to spend more and more time drawing up plans, writing reports, and dealing with management.

When I was young it was fashionable to laugh at the cumbersome Russian bureaucracy where efficiency was lost in endless 'red tape'. Today we cannot laugh because we have the same problem. Big operations are notoriously difficult to manage. In the 'good old days' the fundamental researcher picked a problem, carried out experiments, and based on the results, decided what to do next. If he was intelligent and lucky he learned some new scientific truth.

Usually he did not worry too much about immediate practical applications, but if the research was in a relevant area, the gradual increase in understanding inevitably led to useful advances. Thus, for example, increased knowledge of insect physiology, habits and life cycles should finally lead to better means of controlling insect pests. But today the researcher must write a proposal, draw up a research plan, and identify the expected results as 'milestones' to be met at preset deadlines. Funding is dependent on real or perceived military relevance. If the project is funded, both the scientist and his managers will be rated according to the number of 'milestones' met on schedule. The managers rarely assign problems or suggest ways of solving problems because usually they are not scientists and have only a superficial understanding of scientific research. Their role is to select projects that will 'sell' and to defend them in Washington and then to keep an eye on progress through frequent written 'progress reports' and oral reviews. Inevitably this system favors pedestrian research with easily met objectives over more imaginative and risky difficult science. It also favors optimistic write-ups with exaggerated claims of relevance. Such an atmosphere is not attractive to a creative scientist.

If a scientist does not come up with an acceptable project this does not necessarily mean his departure from the laboratory. The rules are: (1) everyone must be funded from a project, and (2) layoffs of employees must be avoided. Therefore those project leaders with successful proposals that have achieved good funding and who dreamed of hiring bright young staff are more likely to find themselves saddled with an unfortunate who has not achieved funding of his own. All too often the new addition possesses inappropriate skills, is unhappy with his new assignment, and has probably through long tenure achieved a relatively high grade and pay, which is retained. Through no fault of his own he is an expensive and not very valuable addition to the group and definitely detrimental to its morale.

I have described what tight management does to a research laboratory. You are thinking that perhaps it is not quite so serious in development or product laboratories or in production facilities. This is true only if management is competent and really understands the process being managed. Unfortunately today many managers are selected not for their technical competence but for their managerial skills. They are graduates of business courses, systems analysts, MBAs. When scientists or engineers move into management, their on the job training consists of courses on how to manage with much emphasis on subjects such as equal opportunity or sexual harassment. It is the bench people who take courses in new scientific developments, mathematics, instrumentation, and machine tools. So the manager is frequently dependent on the competence of his work force and he is not always an adequate judge of that competence. Procurement officers are dependent on the competence of their suppliers and in addition are subject to great pressures from contractors and politicians. In January 1986 the managements of NASA and Morton Thiokol overruled the objections of the engineers and ordered the fatal launch of the Challenger. This is a recent change. Back in World War II, General Leslie Grove was there to give Robert Oppenheimer whatever he asked for to develop and produce the atom bomb. General Grove handled the Administration headaches, Robert Oppenheimer made the decisions.

Military life can be quite comfortable in peacetime. After World War II many of the military were dispersed around the world as occupation forces in defeated countries or garrisons in allied nations or various American outposts. Travel expenses, medical care, and family housing were provided and shopping in military PXs (post exchange) and commissaries shielded the troops from postwar shortages and inflation. Overseas troops were regularly rotated home to schools, training camps, or headquarters duty. Safe behind the atom bomb, at first no one had to fight. When the dirty little wars, Korea and Vietnam, came along most of the fighting and dying was done by young recruits. Short tours of war zone duty by the officers enhanced their military careers. In these wars we avoided the use of nuclear weapons, nerve gas, and other terrible new weapons and fought in the old-fashioned unsophisticated style because of the type of terrain and the nature of the enemy and because we had to be careful not to provoke Russia or China too much.

Now the draft has ended and the military is dependent on volunteers. 'Volunteer' is a misnomer; our army is recruited. When the draft ended pay was raised and working conditions were improved. Enlisted personnel no longer do KP (kitchen patrol) duty and the old 7-day week, 24-h duty concept exists only under combat. Most soldiers today have a 5-day week and a 7 to 8 h day like the rest of us, but they still retain the generous leave provisions of the old system. We get a good supply of young officers. The Military Academies give a free education and the Armed Services will support young people in colleges or universities in return for a few years of service. The young officer has responsibility and authority, albeit in a small domain. Promotion in the junior ranks is almost automatic. Many of the young officers leave the service as soon as their obligation is completed. Some went in frankly for the free education and a few years of responsible duty, which looks good on a resume. Others are disillusioned by the rigidities and inefficiencies of the system and feel helpless to change it. The ones who stay fit in more easily, accepting the drawbacks and enjoying the advantages. This selection process has been going on for 20 years now, and it has led to a stodgy, complacent, and unimaginative cadre of senior officers. Enlisted personnel tend to be poor and uneducated and their duties are less agreeable. But it is not a bad life and is a good way to escape from disadvantage. Here too, turnover is high. After one tour of duty the more energetic and ambitious return to civilian life. The others move up, become non-commissioned officers and wait out their 20 years to retirement.

Could this army really fight and win an all-out war? Viewing them from my small laboratory, I am skeptical. The system is too big and responsibility too diffuse and too many decisions are based on politics. It is too easy to hide mistakes by classifying them. Real expertise is lacking. The commanding officers do not understand what we are doing. They read our proposals and listen to our pitch but they do not ask any questions that reveal any understanding of what we are proposing or what the army needs. They convey our requests to Washington and monitor how we spend the money we get. They worry a lot about equal opportunity, inappropriate travel, legal trivia, security, and appearances and, most important, getting all reports and other 'pieces of paper' to the required spot on time. They are rotated every 18 to 24 months to a new post. A major objective is a clean record here. If anything negative does surface they try to sweep it under the rug until after their departure. A few months after they leave we have forgotten them and they have forgotten us. In Washington much effort is spent on perpetuating the system and jockeying for desirable positions in it. Each branch of the service: Army, Navy, Marine Corps, Air Force, is competing with the others for money, and manpower, and possession of the fanciest (and most expensive) weapons.

In the field, no one wants to go out and have someone shoot at him. Rather they hope for ever more elaborate weaponry so that no one will dare to attack us. Most of the weapons have not been tested and are not even testable under battlefield conditions. The enlisted men who will have to operate and maintain them lack technical background and training. The officers are not much better. We neglect the simpler conventional weapons and build ever greater stocks of nuclear weapons even though their use is really unthinkable. They are today's Maginot line. Much of today's military planning is based on wishful thinking and untested gadgetry. It is quite appropriate that a lot of this planning is based on simulation from war games. Ideas that would be dubious in science fiction are enthusiastically proposed and supported. This is how we have been led down too many primrose paths to disaster: the Bay of Pigs, the POW rescue in North Vietnam, the desert mission to rescue the Iranian hostages, our dogged support of failed regimes, our brief landing in Lebanon and precipitate withdrawal, etc., etc. When the machines fail or our bluff is called we run away. My worst nightmare is that someday, when everything goes wrong in one of these adventures, we will use nuclear weapons to extricate ourselves and to conceal our folly. And our folly is compounded because so many of these operations are covert and details are kept secret not only from the enemy but from most of the American people including much of the government. Thus there is no chance for responsible debate on feasibility or desirability of these projects.

Even when foolish statements are made there is great reluctance to challenge them or to retract them. In the late 1970s a few people decided that the Russians were spraying terrible mycotoxins ('yellow rain' = fungal toxins) on innocent civilians in Southeast Asia even though mycotoxins lack the requisite qualities for chemical warfare agents (they are slow to act and are toxic only when ingested in relatively high quantity) and only one laboratory was able to detect any significant level of mycotoxin in any of the samples submitted for analysis. Much propaganda and many protests were issued even after investigators concluded that 'yellow rain' was probably bee feces. I suspect that the original theory dawned in some colonel's mind when he noticed yellow spots on the foliage in an area after an attack, perhaps one where tear gas or other crowd control agents had been used. His subordinates were good yes men who went along, higher-ups in the military and state departments, eager for evidence of the depravity of the Russians and fascinated by the high technology aspects of the accusations, made a big thing of it, and the scientists who should have protested kept quiet either in hopes of large research grants or in fear of being labeled subversive spoil sports. For a few years microbiologists and chemists in our laboratory were heavily involved in mycotoxin research. It is revealing that there were never any safety restrictions on handling the mycotoxins. Gradually these projects quietly expired and were not renewed. Yet no government body ever issued any paper or report withdrawing or qualifying the original accusations.

In March 1983, President Reagan proposed the Strategic Defense Initiative and promised that it would provide an impenetrable protective shield for the American population against incoming nuclear weapons. In 1987 the Administration is pushing for early deployment despite the findings of a 15-member panel of the American Physical Society that 'even in the best of circumstances a decade or more of intensive research would be required just to provide the technical knowledge needed for an informed decision about the potential effectiveness and survivability of directed energy weapons'. One may also wonder how, when we cannot seal our borders against millions of illegal aliens, we plan to keep out weapons smuggled in by ship, motor vehicle, low flying aircraft, or even hand-carried package. The goal of protecting people has been quietly dropped except by a few diehards, the real objective now is to protect important missile launching sites.

Even if the military can fight and the weapons do not fail, we no longer have the financial and industrial base to sustain a war. Our steel industry is moribund and our automobile and machine tool industries in decline. A recent defense study concluded that the United States was rapidly losing manufacturing capability in integrated circuits and that the quality of American chips technology was steadily deteriorating relative to the Japanese. Our Vietnam adventure and recent peacetime military buildup have been paid for by enormous budget deficits. In 1980 our national debt was 1 trillion dollars. Today, in 1987, that debt is over 2 trillion dollars and is expected to reach 3 trillion before 1990. So like some improvident third world country we borrow just to pay the interest on the debt, and more and more of these payments go to overseas investors who invest heavily in our treasury bonds. In 1980 we were the largest net creditor in the world (to the tune of 140 billion dollars), now our net foreign debt is about 200 billion and we are the world's largest net debtor. More and more of the goods that we buy are produced in other countries. Once the United States was rightly admired for its technological capabilities, its ability to invent, manufacture machines, and to produce goods. This productive capability was our major contribution to the World War II victory. Today we are increasingly a nation of managers, service industries, and financial manipulators. A young person who aspires to be rich heads for Wall Street and engages in transactions that produce money, not things. Meantime the Japanese own major hotels on Waikiki Beach, banks in California, office buildings in New York, and a growing number of factories in the Midwest. If a Rip Van Winkle woke up today from a nap that began in World War II, he might think the Japanese had won that war.

So who is to blame for our gloomy predicament? My assessment is Pogo's 'I have seen the enemy and they is us'. We have elected and re-elected the politicians and supported the policies that led us to the present fiasco. The result is that more and more of our resources go to support the military and its satellite aerospace industry but we are unwilling to pay the price now in higher taxes and/or reduced expenditures in the civilian sector. So it all goes on the cuff to be paid for in the future by a reduced American standard of living. Many of our best scientists and engineers have been drawn by attractive salaries and support, and exciting intellectual challenge to work on the sophisticated systems for weapons and space vehicles. Cost has been no object and quality control is lax. Meantime countries like West Germany and Japan, where defense budgets are minuscule, have concentrated on efficient production of high quality consumer items. Sooner or later the product will speak for itself and the consumer will purchase the best. So these countries have flourishing economies while the economies of Britain, Russia, and the United States, the World War II victors, are staggering.

Is it too late to reverse direction? It would require a willingness to admit mistakes and to sacrifice that is not congenial to the American people. But perhaps if we admit that without reform we are headed for military disaster and financial collapse that will force painful changes on us, we can summon up the resolve to control and improve our destiny. The first required change will be in attitude. We are not superior to other people (after all, we are a nation of immigrants from those other countries) and our problems are not due to Russian spies and Japanese trade barriers. Secondly, we must restore our morality and our patriotism. We will progress only if our adults work hard and support our industry and our government because they deserve it, and if our young people receive a good and rigorous education. This does not mean throwing money at the problems. That has been our solution in the past and most of the money has ended up being spent on more management. We need better teachers, not more high paid administrative superstructure. We need more people who do things and fewer people who manage them. The managers we do have should be competent and accountable. Thirdly, the military and intelligence establishments need drastic reduction and reform. The function of the military is to protect the country, not to provide cushy careers for officers and profits for defense contractors. Perhaps we should adopt a system where all young people are called up for intensive training for a year or so and then brought back every 2 or 3 years for a brief refresher course. We should look for officers who have intelligence and initiative and encourage them to speak out and to make suggestions. We should greatly reduce overseas bases and meddling in foreign governments. We should also greatly reduce secrecy and covert operations. These hurt us far more than the enemy because they conceal and perpetuate folly and immorality. Let our embassy in Moscow remain bugged and let the Russians hear every word that is said there. Their KGB files will be so cluttered with trivia that they will never find the occasional tidbit of value. If they understand us better perhaps they will like us better. Finally we should cease production and stockpiling of weapons that would destroy civilization and pursue genuine arms control with the Russians.

Does all this sound too Utopian? Do you protest that reduction of military forces and of weapons procurement would result in widespread unemployment and recession? No doubt there would be painful readjustments; that is what I meant by a willingness to sacrifice. But reduction in military expenditures would free funds to restore roads, bridges, and public transport, and to clean up polluted air, water, and soil. It could lead to a reinvigoration of the civilian economy. All those defense contractors and ex-soldiers would need to do something! Do you fear the Russians would take advantage? My mother used to say that it takes two to start a fight and to keep it going. The Russians are as eager for peace as we are. They initiated the 1960s moratorium on nuclear testing, and they had a moratorium on nuclear testing from mid 1985 through January 1987. During that period we set off 24 tests. I do not advocate total disarmament and I do support universal military training. I believe that a leaner, more efficient military establishment and a restriction of military adventures to genuine threats against our own territory would increase our security. I also believe that true strength depends on a clear conscience and a healthy civilian economy.

So here you have it, a view of the military and the nation as seen through the naive and trusting eyes of youth, the gradual disillusionment and reality of middle age, and finally the more cynical but still optimistic view of old age. Of course we were not that pure and good 50 years ago and of course the international situation today is complex and difficult! That still leaves much truth in what I am saying and I do believe the perceptions of many other citizens are progressing to the same conclusions.

